# Supplementary material for: Catalytic inhibition of H3K9me2 writers disturbs epigenetic marks during bovine nuclear reprogramming
Source: Sci Rep. 2020 Jul 13;10:11493. doi: 10.1038/s41598-020-67733-9 (PMC7359371; doi:10.1038/s41598-020-67733-9)
Supplement: Supplementary file 1 — Supplementary file1 [file 41598_2020_67733_MOESM1_ESM.pdf]

Title: Catalytic inhibition of H3K9me2 writers disturbs epigenetic marks during bovine nuclear reprogramming.

Atuhors: [1,3,4]Rafael Vilar Sampaio*, [1,4]Juliano Rodrigues Sangalli, [1]Tiago Henrique Camara De Bem, [1]Dewison Ricardo Ambrizi, [1]Maite del Collado, [1] Alessandra Bridi, [1]Ana Clara Faquineli Cavalcante Mendes de Ávila, [2]Carolina Habermann Macabelli, [1]Lilian de Jesus Oliveira, [1]Juliano Coelho da Silveira, [2]Marcos Roberto Chiaratti, [1]Felipe Perecin,[1]Fabiana Fernandes Bressan; [3]Lawrence Charles Smith, [4]Pablo Juan Ross, [1]Flavio Vieira Meirelles.

[1]Departamento de Medicina Veterinária, Faculdade de Zootecnia e Engenharia de Alimentos, Universidade de São Paulo, Pirassununga, SP, Brazil. [2]Departamento de Genética e Evolução, Universidade Federal de São Carlos, São Carlos, SP, Brazil. [3] Université de Montréal, Faculté de médecine vétérinaire, 4.[4]Department of Animal Science, University of California Davis, USA.

***sampaiorv@gmail.com**

A B

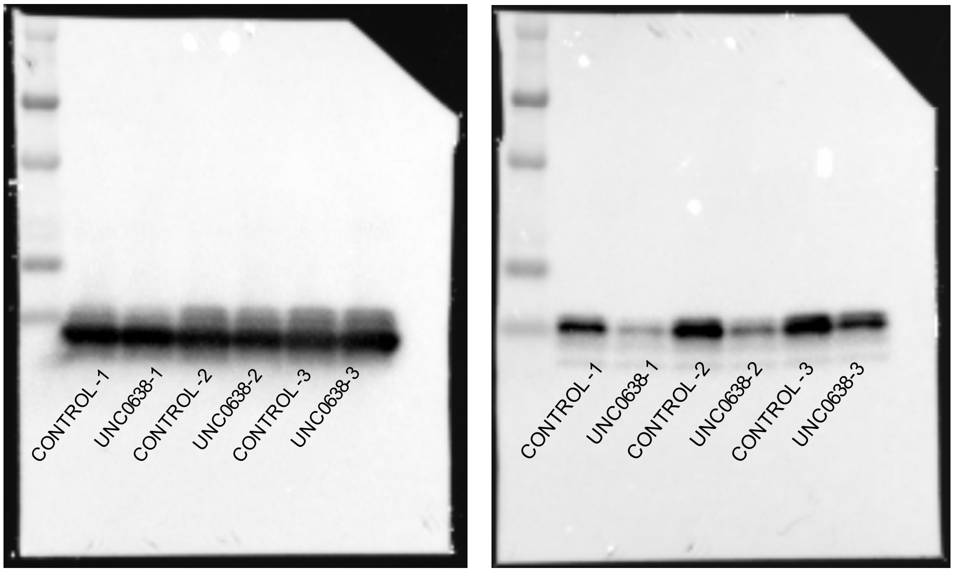

C **D**

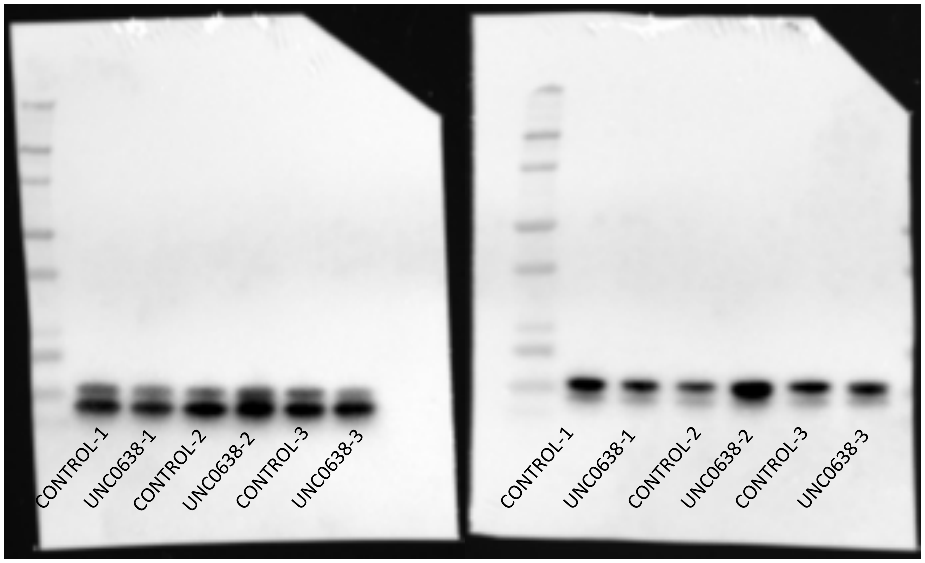

Supplemental Figure 1 Full immunoblots from data presented on Figure 1. (**A**)-Total histone 3. 1 (**B**) Levels of H3K9me2, (**C**)-Total histone 3, and (**D**)- Levels of H3K9me3.

Supplemental table 1. Primer sequences used in Real Time qPCR.

Accession number Primer Sequences (5’-3’) Product (bp) AT (°C)

Gene Symbol	Accession number or reference	Primer Sequences (5’-3’	Product (bp)	AT (°C)
PPIA	XM_010804358.3	F: GGTCCTGGCATCTTGTCCAT	94	60
		R: TGCCATCCAACCACTCAGTCT		
RPL15	XM_005226176.2	F: CAAACGCCCAGTTCCTAAGG	76	60
		R: TCGAGCAAACTTGAGCTGGTT		
DNTM1	XM_015471995.2	F: GCAGTACCAGCCCATCCT	64	60
		R: GCGGGCAGCCACCAA		
DNMT3A	NM_001206502.2	F: GCTCATGTGTGGGAACAACAATT	64	60
		R:CACCAAGAGATCCACACATTCCA		
DNMT3b	NM_181813.2	F: GTCCTTCCACCCTCTCTTTGAG	85	60
		R:GTCGTCGTCGTACATGTAGAAGA		

TET1	XM_024986940.1	F: TGCCTACTTGCAACTGTCTTGATC<br>R: TCTATCCTTACTGCATTTCCTTTTTG	145	60
TET2	XM_005207682.4	F: GGATACACCTGTCAAGACTCAGTATGA<br>R: GGACCTGCTCCTAGATGGGTATAA	105	60
TET3	XM_005212473.4	F: TGCGGCCTCAACGATGA<br>R: GGAACAACCGAAGGAGAAGGA	84	60
EHMT1	XM_024998813.1	F:CAAGAGGAAGGGGAAGCCTG<br>R:AACCTAACACAGAGCAGGGC	92	60
EHMT2	XM_005223636.3	F: GTTTGCCCTCCAGCTCAAC<br>R: GTCCACACCATTGACACAGG	133	60
